# Comparative Evaluation of Lipofectamine and Dendrimer for Transfection of Short RNA Into Human T47D and MCF-10A Cell Lines

**DOI:** 10.34172/apb.2023.022

**Published:** 2022-04-30

**Authors:** Zohreh Jahanafrooz, Behnaz Bakhshandeh, Erfan Shirzadi

**Affiliations:** ^1^Department of Biology, Faculty of Sciences, University of Maragheh, Maragheh, Iran.; ^2^Department of Biotechnology, College of Science, University of Tehran, Tehran, Iran.; ^3^Institute of Chemical Sciences and Engineering, Swiss Federal Institute of Technology Lausanne, École Polytechnique Fédérale de Lausanne, Switzerland.

**Keywords:** Short RNAs, T47D, MCF10A, Lipofectamine, PAMAM dendrimer

## Abstract

***Purpose:*** Non-viral transfection approaches are extensively used in cancer therapy. The future of cancer therapy lies on targeted and efficient drug/gene delivery. The aim of this study was to determine the transfection yields of two commercially available transfection reagents (i.e. Lipofectamine 2000, as a cationic lipid and PAMAM G5, as a cationic dendrimer) in two breast cell lines: cancerous cells (T47D) and non-cancerous ones (MCF-10A).

***Methods:*** We investigated the efficiencies of Lipofectamine 2000 and PAMAM G5 for transfection/delivery of a labeled short RNA into T47D and MCF-10A. In addition to microscopic assessments, the cellular uptakes of the complexes (fluorescein tagged-scrambled RNA with Lipofectamine or PAMAM dendrimer) were quantified by flow cytometry. Furthermore, the safety of the mentioned reagents was assessed by measuring cell necrosis through the cellular PI uptake.

***Results:*** Our results showed significantly better efficiencies of Lipofectamine compared to PAMAM dendrimer for short RNA transfection in both cell types. On the other hand, MCF-10A resisted more than T47D to the toxicity of higher concentrations of the transfection reagents.

***Conclusion:*** Altogether, our research demonstrated a route for comprehensive epigenetic modification of cancer cells and depicted an approach to efficient drug delivery, which eventually improves both short RNA-based biopharmaceutical industry and non-viral strategies in epigenetic therapy.

## Introduction

 Short RNAs can influence different hallmarks of breast cancer such as proliferation, apoptosis resistance, invasion/migration, and angiogenesis/new vessel formation. Efficacy and feasibility of short RNA therapies has been confirmed in preclinical attempts.^1-3^ On the other hand, the onset of viral diseases and emerging nucleic acid-based drugs, which usually have better effects than protein-based drugs, has led pharmacists and biotechnologists to produce more efficient and safer nucleic acid carriers into the cells. Because of the negative charge of the plasma membrane and the negative charge of nucleic acids, the delivery of nucleic acid-based drugs and vaccines is inefficient. Recently, new methods for short RNA transfection offer ways for inactivation of target genes and also analyses of gene function.^4-7^ Production of genetically modified cells through delivery of foreign nucleic acids into the cells is called transfection, which includes biologically, physically, and chemically mediated methods with various benefits for particular applications.^8^ Due to the complications of the traditional delivery methods, researchers are looking for new methods and systems to more efficient delivery. Several factors such as kind of the nucleic acid, cell types and medium conditions have an influence on transfection efficiency. An appropriate transfection method has both high transfection efficiency and low cytotoxicity.^9,10^ Virus-mediated transfection or transduction is commonly utilized in clinical researches. Although viral vector is an efficient and promising delivery method, it has some disadvantages, including viral recombination, limited size of DNA, host immunological response, and some other possible undesired effects such as off-target or oncogenic effect.^10,11^ Physical transfection methods, including electroporation, biolistic particle delivery or micro-projectile bombardment, microinjection and laser-based transfection, require various physical tools.^12^ Lipidoids, cationic polymers, carbon nanotubes, cell-penetrating peptides and cationic protein–antibody fusions are used in chemical transfection methods.^8,13^

 Liposomes are one of the most notable candidates for drug delivery. Liposomes are spherical vesicles that have an aqueous nucleus surrounded by one or more phospholipid layers and are usually divided according to size (small, large and very large), the number of bilayers (monolayer and multilayer) and the charge of phospholipids (neutral, anionic or cationic). Cationic liposomes are much more effective and safer, and previous studies have shown that they improve the efficiency of gene transfer compared to other techniques. Cationic lipids apply chemical vectors to condense nucleic acid through ionic interaction. Lipid subunits of Lipofectamine reagent can form liposomes in an aqueous environment and entrap oligonucleotide.^14^ Lipofectamine 2000 is a cationic liposome composed of both poly-cationic and natural lipids. Lipofectamine 2000 compared to the old prototype of Lipofectamine series such as Lipofectin is less toxic, and can transfect a wide variety of both adherent and suspend cells in the absence or presence of FBS. Lipofectamine 3000 is even more improved prototype of Lipofectamine.^15^ In one recent study, five commonly used transfection reagents, including Lipofectamine 3000, Lipofectamine 2000, Fugene, RNAiMAX and Lipofectin, were comprehensively analyzed in ten cell lines. According their results, Lipofectamine 3000, Fugene and RNAiMAX showed high transfection efficacy with lower toxicity compared to Lipofectamine 2000. The mentioned study also showed that both transfection efficacy and toxicity of the transfection reagents are cell type dependent.^16^

 PAMAM G5 (polyamidoamine), as a cationic dendrimer, has homogenous and nanometric size and globular shape with various changeable surface functional groups and wide molecular weight range for drug and gene delivery. Over the last decades, there have been many promising results for applications of dendrimers in biology or medicine. PAMAM dendrimer suggested as a promising nonviral gene carrier in cancer therapy. PAMAM dendrimer are stable and hardly oxidized compared to cationic liposomes.^17,18^ The unique characteristic of dendrimer makes the dendrimer more favorable, in which drugs have been attached or encapsulated to the peripheral active amine groups of dendrimer that modulate its solubility and cytotoxicity.^19,20^ These highly branched macromolecules are effective carriers for drug and gene delivery in cancer therapy. In a recent study, PAMAM G3 was cross-linked with 4,4′-dithiodibutryic acid (DA) to form nanoclusters (NCs). The synthesized G3-DA NCs increased 2.3 and 2.1 times gene transfection to cancer cells compared to the PAMAM G3 and PAMAM G5, respectively, under the same conditions.^21^

 Hydrocortisone is a glucocorticoid hormone, which stimulates cellular growth in cell culture.^22^ Hydrocortisone is one of the ingredients in MCF-10A cell culture. Since hydrocortisone can interact with Lipofectamine and interfere in transfection process,^23^ the T47D cells were also cultured with hydrocortisone, to evaluate the possible effect of hydrocortisone on transfection efficiency. Our team reported the transfection efficiencies and toxicity of DOTAP (a liposome) and PAMAM G5 in stem cells previously.^4,14^ In the present study we evaluated transfection efficiency and toxicity of PAMAM G5 dendrimer and Lipofectamine in T47D (breast cancer cell line) and MCF-10A (non-malignant breast cell line) cells. For this purpose, scrambled FITC-conjugated RNA as a type of short RNA was transfected into mentioned cell lines with both transfection reagents. In addition, the necrosis of both cells was evaluated to determine the susceptibility of cells to mentioned reagents.

## Materials and Methods

###  Cell culture 

 T47D cells (human ductal breast epithelial tumor cell line) were cultured in RPMI-1640 containing 10% fetal bovine serum (FBS) and 1% Penicillin/streptomycin antibiotics (Gibco, UK) incubated at 37°C in 5% CO2 under 95% humidity. MCF-10A cells (non-cancerous human breast epithelial cell line) were cultured in DMEM/F12 (Invitrogen, USA), supplemented with 15% FBS, 10 μg/ml insulin (Sigma-Aldrich, USA), 100 μg/mL hydrocortisone (Sigma-Aldrich Chemie GmbH, Germany), and 1% pen/strep (Gibco, UK) incubated at 37°C in 5% CO2 under 95% humidity. The media was exchanged every other day.

###  Size or zeta potential evaluation of the complexes of Lipofectamine or PAMAM/RNA & Cell Transfection

 Polyamidoamine dendrimer generation five or PAMAM G5 was synthesized and characterized according the method described in our previous work (i.e., synthesis was performed via iterative reactions of Michael addition of methyl acrylate to the ethylenediamine for half generations.^14^ Then exhaustive amidation of half generations by high excess methanolic ethylenediamine in the next step for full generations. The final product was characterized by ^1^H NMR, FT-IR and gel permeation chromatography). As the same of our previous report, Malvern Nanosizer ZN series was used for the size or zeta potential evaluation of the complexes, according to the manufacturer’s instructions (Malvern Instruments Ltd, Worcestershire, UK).^14^ The size of PAMAM/RNA complexes was independent of N/P ratios. Zeta potential of Lipofectamine and PAMAM/RNA complexes (around 20–25 mV at N/Ps of 5 and higher) reached a steady state charge. Our synthesized PAMAM G5 has 128 primary amino groups at their surface with 28826 Da.^14^ 25 pmol of scrambled short RNA (miRCURY LNA^TM^ RNA, 5nmol, 3ʹ-fluorescein labeled, Exiqon, Woburn, MA, USA) with 0.4 μL and 0.75 μL of Lipofectamine 2000 (Invitrogen, US) or with N/P ratios (nitrogen in PAMAM/phosphate in scrambled miRs) of 10, 20, and 40 of polyamidoamine (PAMAM G5 dendrimer^14^) were combined, gently vortexed for few seconds, and incubated for the appropriate time (according to specification of different reagents and laboratory condition: 20 minute in this study) to allow formation of complexes, as in the similar approach reported in previous studies.^14-18^ The 2 × 10^4^ cells were transfected with the prepared complexes in 24-well plates without antibiotics and FBS. After 10-hours incubation, the medium was replaced with fresh medium containing FBS and pen/strep.

###  Transfection evaluation

 Transfection of short RNA in T47D and MCF-10A cells was visualized by fluorescence microscopy (micros MCXI600, Austria). For quantitative analysis of transfection efficiency, the cells were washed twice by PBS, trypsinized and centrifuged for 4 min at 1200 rpm. Paraformaldehyde 4% was used for fixation of re-suspended cells and green cell numbers was analyzed by a 2-beam laser FACSCalibur and CellQuest software.^14^

###  Cell viability assessment 

 To evaluate the effect of Lipofectamine 2000 and PAMAM G5 on viability of T47D and MCF-10A cells, propidium iodide (PI) (Sigma-Aldrich Chemie GmbH, Germany) assay was applied by flow cytometry (BD Biosciences, USA) analysis after 10-hours incubation. Viable cells reject PI but dead cells cannot exhale the dye.^24^

###  Statistical analysis

 All performed experiments were repeated at least three times. Student’s two-tailed *t* test or one-way ANOVA was applied to compare data between two or more groups, respectively. A *P* value < 0.05 was considered statistically significant. The results were depicted as means ± SD.

## Results and Discussion

###  Transfection evaluation by fluorescent microscopy

**Figure 1:**
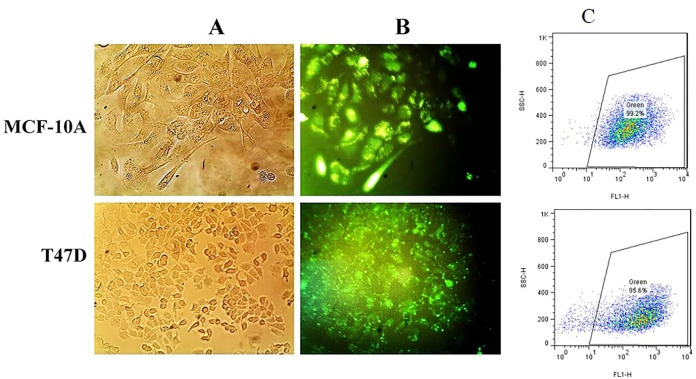
Transfection of FITC-scrambled short RNA into T47D and MCF-10A cells. **(A)** Phase contrast microscopy. **(B)** Fluorescent microscopy. The green spots represent the fluorescent scrambled RNA inside the cytoplasmic Lipofectamine (400X magnification). **(C)** Flow cytometric analysis of transfected cells, percentages (99.2% for MCF-10A and 95.6% for T47D cells) show the transfection efficiencies.

 T47D and MCF-10A cells, transfected with FITC-scrambled RNA and Lipofectamine/PAMAM were subjected to fluorescence microscopy after 10 h incubation. The green fluorescent cells were considered as successful transfected ones (Figure 1). Based on our data, 0.75 μL Lipofectamine per well of 24-well plates would be a better option for transfection in both T47D and MCF-10A cells. Remarkably, there was few numbers of green spots in T47D cells transfected with PAMAM dendrimer; no sign of green spots was detected in PAMAM transfected MCF-10A cells, which caused stopping further analysis in the PAMAM transfected MCF-10A cells.

###  Transfection evaluation by flow cytometry

**Figure 2:**
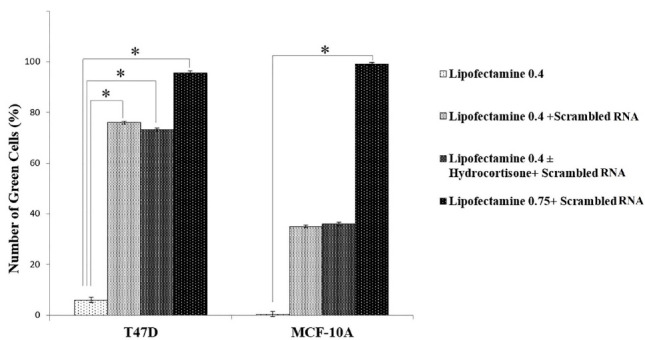
Transfection efficiencies of different quantities of Lipofectamine 2000 and also with (in T47D) or without (in MCF-10A) hydrocortisone. Results are depicted as mean ± SD. (*P* value = 0.01053, 0.0238, and 0.0071 in T47D cells and 0.0038 in MCF-10A cells) * *P* < 0.05 was considered significant difference compared to control (Lipofectamine 0.4 µL per 24 well).

**Figure 3:**
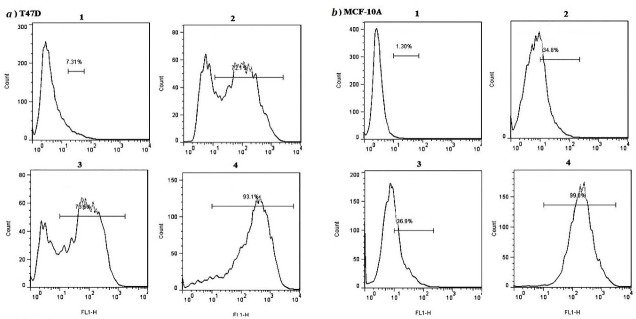
Flow cytometry analysis of transfected T47D **(a)** and MCF-10A **(b)** cells. Percentages present the transfection efficiencies. **1)** Lipofectamine 0.4, **2)** Lipofectamine 0.4 + Scrambled short RNA, **3A)** Lipofectamine 0.4 + Scrambled short RNA + Hydrocortisone, **3B)** Lipofectamine 0.4 + Scrambled short RNA - Hydrocortisone, and **4)** Lipofectamine 0.75 + Scrambled short RNA. Increase in Lipofectamine 2000 concentrations resulted in higher transfection efficiency.

**Figure 4:**
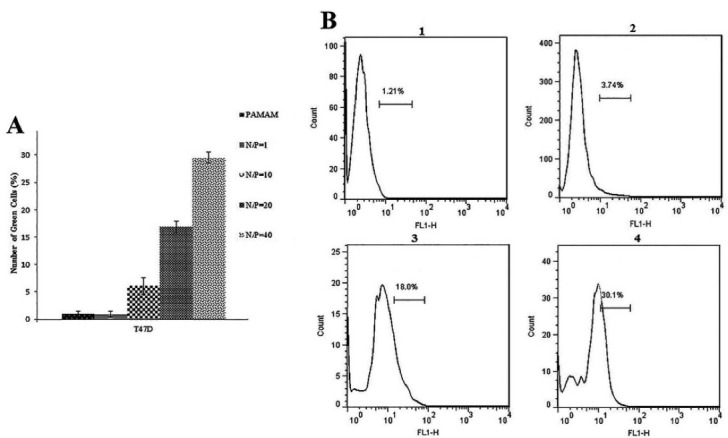
Transfection efficiencies in different N/P ratios of PAMAM G5 in T47D cells (**A** and **B**). Results are expressed as mean, and compared to PAMAM G5 as control (Error bars: ± 1 S.D.). **1)** PAMAM G5 without scrambled short RNA (concentration of PAMAM was equal to the case of N/P = 40) and **2)** N/P = 10, **3)** N/P = 20, **4)** N/P = 40, (N = number of nitrogen of PAMAM and P = number of phosphate of scrambled).

 Quantitative evaluation of transfection was performed by flow cytometry. In our flow cytometry results the transfection reagent treated samples regarded as the control because untreated sample or blank ones were as the same of the controls. As shown in Figure 2, using 0.4 μL Lipofectamine, transfection efficiencies were 76% ± 0.6 and 36 ± 0.7 in T47D and MCF-10A cells, respectively. Furtherance, in the presence of hydrocortisone, transfection efficiencies were 73.2% ± 0.5 for T47D cells and 35% ± 0.6 for MCF-10A; so hydrocortisone had no significant effect (*P* value = 0.620528 in T47D and 0.762175 in MCF-10A) on transfection efficiency in these cells. Application of 0.75μL Lipofectamine increased the transfection efficiencies in MCF-10A cells to 99.2 ± 0.2 and in T47D cells to 95.6 ± 0.8 (Figures 1, 2, 3, and Figure S1). In congruence with microscopic data, transfection efficiency of PAMAM dendrimer in both cell types was very low (Figure 4). 0.4 μL Lipofectamine would be a better option for transfection in T47D because of the high transfection yield and the negligible cytotoxicity. However, transfection efficiency of 0.4 μL of Lipofectamine in MCF-10A cells was very low. Lipofectamine entrance to the cells is mostly dependent on endocytosis; therefore, fluidity of cell membrane and protein composition can be suggested as some determining factors for endocytosis and subsequently transfection efficiency by Lipofectamine liposomes.^25^ Differences in cell membrane properties between cancerous and normal cells can explain the abovementioned results.^26^ Lipofectamine 2000 demonstrated the transfection efficiency of 33.29% in MCF-7 breast cancer cells, 22.21% in SHSY5Y neuroblastoma cells, and 7.89% in HL60 promyelocytic leukemia cells. Therefore, cancer cells can also display strong resistance to some transfection reagents such as Lipofectamine 2000.^16^

 PAMAM G5 dendrimer showed low yield of transfection in T47D cells while no noticeable transfection in MCF-10A cells in any molar ratios (N/P from 1 to 40) was detected; therefore, dendrimer is not suggested as a transfection agent for any of mentioned cell types. Notably, previously we reported PAMAM G5 at lower N/P ratios (0.5, 1, and 2.5) as a successful transfection reagent for mouse embryonic stem cells (mESCs).^4,14^ Overall, the yield of transfection in stem cells was closer to cancerous cells than non-cancerous ones. Again, this difference in transfection yields between above noted cells emphasizes on the influence of cell types and their different cell membrane characteristics on transfection yields. As a side note, there are some common surface proteins and signaling pathways between cancer cells and stem cells which could justify the transfection similarity between a stem cell and a cancer cell.^27^

###  Effect of transfection reagents on cell viability

**Figure 5:**
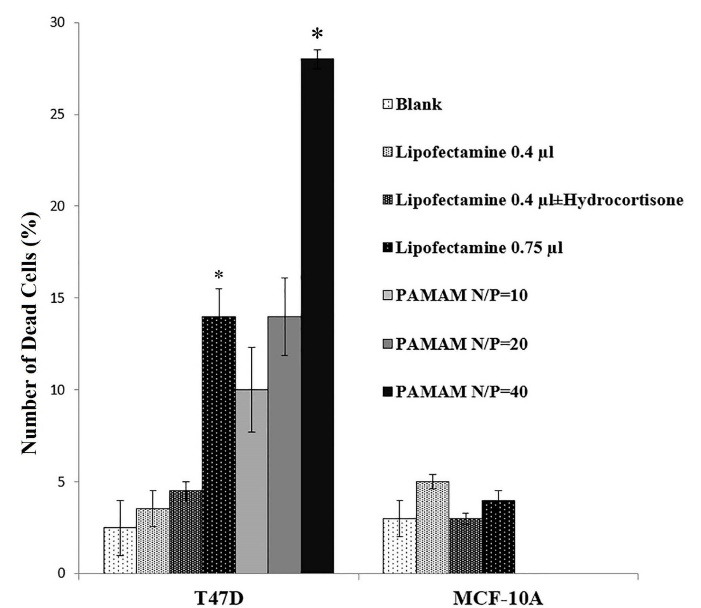
Effect of Lipofectamine 2000 and PAMAM G5 on cell viability in T47D and MCF-10A (Because of low transfection efficiency in MCF-10A by PAMAM G5, viability assay was not performed). Results are expressed as mean ± SD. * *P* < 0.05 was considered significant difference compared to blank.

**Figure 6:**
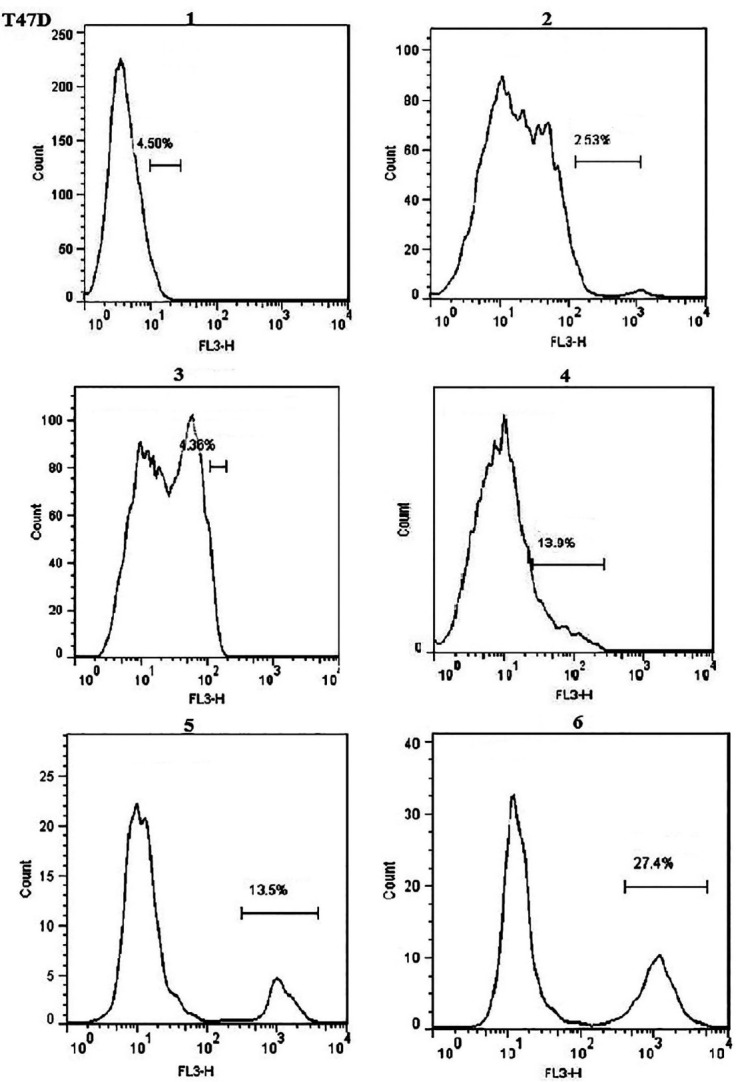
Flow cytometry diagrams of Lipofectamine 2000 and PAMAM G5 effect on cell viability in T47D. **1)** Control, **2)** Lipofectamine 0.4 + Scrambled short RNA, **3)** Lipofectamine 0.4 + Scrambled short RNA + Hydrocortisone, **4)** Lipofectamine 0.75 + Scrambled short RNA, **5)** PAMAM N/P = 20, and **6)** PAMAM N/P = 40.

**Figure 7:**
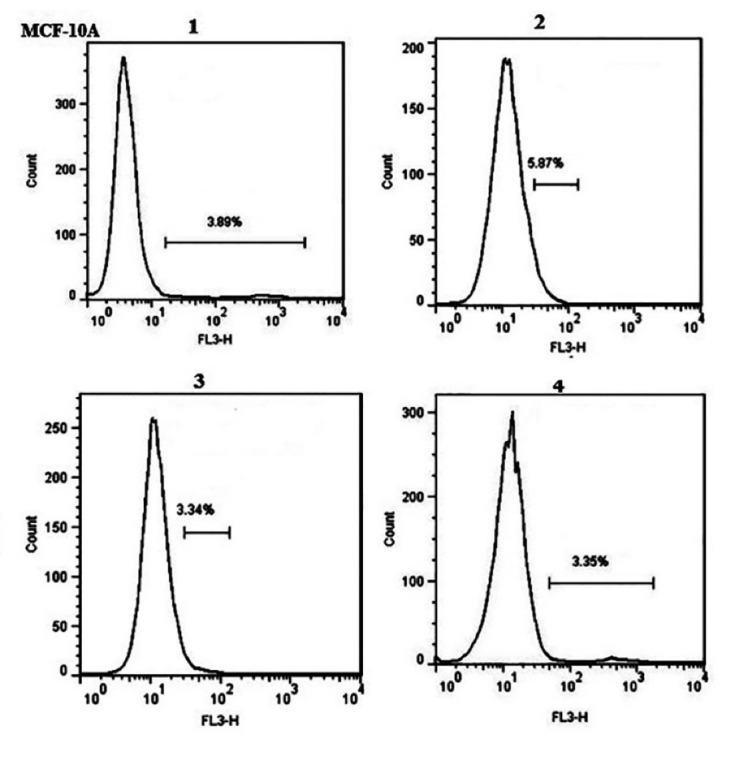
Flow cytometry diagrams of Lipofectamine 2000 effect on viability of MCF-10A cells. **1)** Blank media, **2)** Lipofectamine 0.4 + Scrambled short RNA, **3)** Lipofectamine 0.4 + Scrambled short RNA - Hydrocortisone, and **4)** Lipofectamine 0.75 + Scrambled short RNA.

 In the case of transfection by Lipofectamine, higher reagent concentrations improved transfection efficiency significantly in T47D and MCF-10A cells while cell viability significantly (*P* value = 0.034967) decreased in T47D cells compared to blank (Figures 5, 6, 7, and Figure S2). This was also reported in other studies in which increase in transfection efficiency associated with enhanced cytotoxicity following higher Lipofectamine 2000 concentrations.^15,23^

 Having no major cytotoxicity of the N/P ratios of 10 and 20 of PAMAM dendrimer, transfection using the N/P ratio = 40 decreased the viability of T47D cells significantly (*P *value = 0.01155) (Figures 5, 6, 7, and Figure S2). Nomani et al showed that cytotoxicity of the complexation of PAMAM dendrimer and RNA was dependent upon the generation and charge ratio of the PAMAM dendrimer; for instance, the toxicity of PAMAM G2 was lower than PAMAM G5. They also reported that cytotoxicity of naked PAMAM G5 and PAMAM G5/short RNA was the same (Table 1).^28^ A suggested solution for decreasing the cytotoxicity of PAMAM and increasing its transfection efficiency is incorporation of cyclic arginine-glycine-aspartic acid (cRGD) peptide to the PAMAM surface. In one study, not only higher transfection efficiency but also less toxicity of PAMAM-cRGD was reported.^29^ Li et al showed that PAMAM-cRGD/siRNA genetically modified spermatogonial stem cells with a hope to cure male infertility.^30^ Overall, in accordance to other studies, Lipofectamine compared to dendrimer, results in more effective delivery with less cytotoxicity in both cancerous (such as T47D and SW480 cell lines) and noncancerous (such as HEK293 and MCF-10A cell lines) cells. Table 1 summarized the cytotoxicity of Lipofectamine or dendrimer/short RNA complex reported by some studies.

**Table 1:** Cytotoxicity of Lipofectamine/dendrimer and short RNA complex reported in some other studies compared to ours

**Cell line**	**Volume in 500 μL**	**Viability (%) compared to blank**	**Ref.**
T47D	0.4 μL Lipofectamine	98.5	This study
	0.75 μL Lipofectamine	88.5	
	PAMAM G5 N/P = 10	92.5	
	PAMAMG5 N/P = 20	88.5	
	PAMAMG5 N/P = 40	74.5	
MCF-10A	0.4 μL Lipofectamine	99
	0.75 μL Lipofectamine	99
T47D	PAMAM G2, G3, G4 and G5	Toxic at the higher generations	^28^
HEK293	1.5 μL Lipofectamine	No apparent toxic effect	^15^
SW480	2 μL Lipofectamine	Without significant toxicity	^31^
Huh-7	2 μL Lipofectamine	75.34	^16^
HEK2		87.29	
MCF-7		92.49	
U87MG		80	

## Concluding remarks

 Altogether, Lipofectamine as a cationic lipid and PAMAM G5 as a cationic polymer are more absorbed by cancer cells; researches confirmed that plasma membrane of cancerous cell is more negatively charged than normal cells and then absorbs positively charged reagents more efficiently.^32^ Moreover, specific lipid composition and higher fluidity of cell membrane in cancer cells causes more tendency to absorb and engulf materials from the extra cellular matrix and environment to change their interior in order to perform more mutations.^33^ Indeed, researches showed cancer cells shed and absorb many microvesicles and microvesicle-like particles such as Lipofectamine in order to inducing metastasis and changes in other cells.^34,35^ In addition to cell type and transfection reagents, structure and chemistry of oligonucleotide are proposed other influencing factors on the cytotoxicity and transfection efficiency.^36^ Overall, our results showed that Lipofectamine had better transfection efficiency than PAMAM dendrimer under the same transfection conditions in both cell types. Given the cell type, T47D cells as a cancerous cell exhibited more transfection efficiency than MCF-10A as a non-cancerous cell with both transfection reagents. More extensive investigations are needed to be performed to clarify the cellular characteristics related to mechanisms influencing the transfection mechanisms and efficient protocols in different cell types.

## Acknowledgments

 The author would like to acknowledge the kind cooperation of Prof. Nasrin Motamed.

## Competing Interests

 The authors declare no conflicts of interest.

## Ethical Approval

 Not applicable.

## Supplementary Files

Supplementary file 1 contains Figures S1-S2.
