# Supplementary material for: Comparative Evaluation of Lipofectamine and Dendrimer for Transfection of Short RNA Into Human T47D and MCF-10A Cell Lines
Source: Adv Pharm Bull. 2022 Apr 30;13(2):385–92. doi: 10.34172/apb.2023.022 (PMC10278214; doi:10.34172/apb.2023.022)
Supplement: Supplementary file 1 — contains Figures S1-S2. [file apb-13-385-s001.pdf]

Adv Pharm Bull, 2023, 13(2), 385-S1
doi: 10.34172/apb.2023.022
https://apb.tbzmed.ac.ir

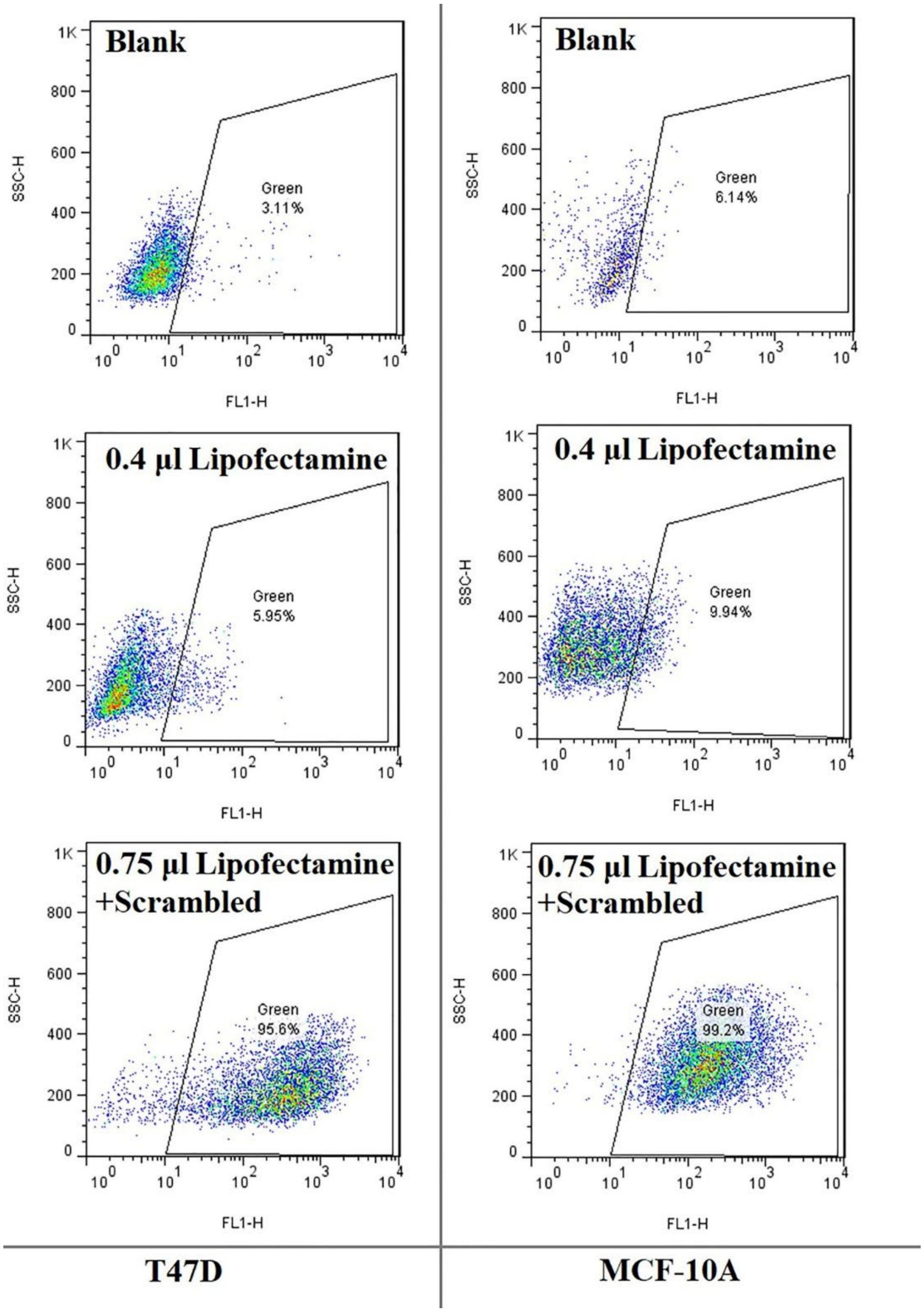

Supplementary Figure 1. Comparison of the scattered plot chart data of flow cytometry analysis in blank, control (only Lipofectamine 2000), and transfected (Lipofectamine 0.75 + Scrambled) T47D and MCF-10A cells. Percentages present the transfection efficiencies. There is no significant difference between blank and control samples.

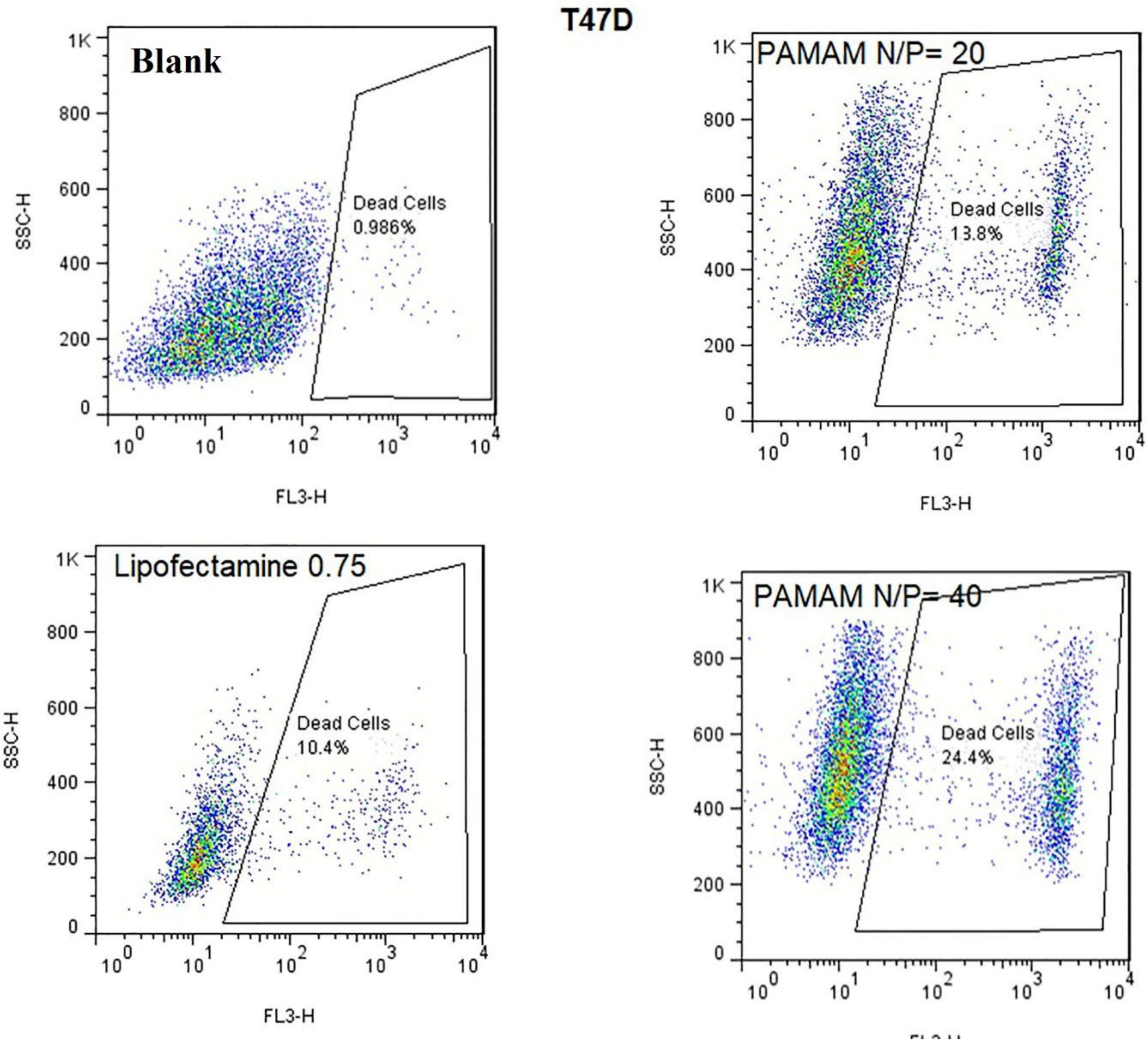

Supplementary Figure 2. Effect of Lipofectamine 2000 and PAMAM G5 on cell viability in T47D cells.
